# Promoting Adherence to Exercise Prescription Using Mobile Health Technology: Lessons Learned from Older Adults

**DOI:** 10.1093/geroni/igaa057.3090

**Published:** 2020-12-16

**Authors:** Zvinka Zlatar, Camille Nebeker

**Affiliations:** University of California, San Diego, La Jolla, California, United States

## Abstract

We will present the implementation approach used to evaluate a novel mobile health (mHealth) exercise intervention designed to improve older adult’s fitness and brain health. A randomized controlled trial design evaluated if the use of mHealth strategies improved walking speed in real world environments to help participants achieve current physical activity guidelines. Cognitively healthy older adults were randomized to a 12-week, unsupervised, exercise prescription condition or to a healthy aging education condition. Participants used a heart rate tracker programmed to provide haptic feedback when they fell outside of their prescribed heart rate target during an exercise session. Participant’s perceptions regarding the mHealth technology device, as well as their feedback regarding the privacy of their data when using mHealth devices will be presented. Recommendations to improve the design of future mHealth exercise trials will be discussed.

